# The Effect of Vitamin D and Its Analogs in Ovarian Cancer

**DOI:** 10.3390/nu14183867

**Published:** 2022-09-18

**Authors:** Karina Piatek, Martin Schepelmann, Enikö Kallay

**Affiliations:** Center for Pathophysiology, Infectiology and Immunology, Institute for Pathophysiology and Allergy Research, Medical University of Vienna, Waehringer Guertel 18-20, 1090 Vienna, Austria

**Keywords:** ovarian cancer, vitamin D, vitamin D analogs

## Abstract

Ovarian cancer is one of the deadliest cancers in women, due to its heterogeneity and usually late diagnosis. The current first-line therapies of debulking surgery and intensive chemotherapy cause debilitating side effects. Therefore, there is an unmet medical need to find new and effective therapies with fewer side effects, or adjuvant therapies, which could reduce the necessary doses of chemotherapeutics. Vitamin D is one of the main regulators of serum calcium and phosphorus homeostasis, but it has also anticancer effects. It induces differentiation and apoptosis, reduces proliferation and metastatic potential of cancer cells. However, doses that would be effective against cancer cause hypercalcemia. For this reason, synthetic and less calcemic analogs have been developed and tested in terms of their anticancer effect. The anticancer role of vitamin D is best understood in colorectal, breast, and prostate cancer and much less research has been done in ovarian cancer. In this review, we thus summarize the studies on the role of vitamin D and its analogs in vitro and in vivo in ovarian cancer models.

## 1. Ovarian Cancer

**Figure 1:**
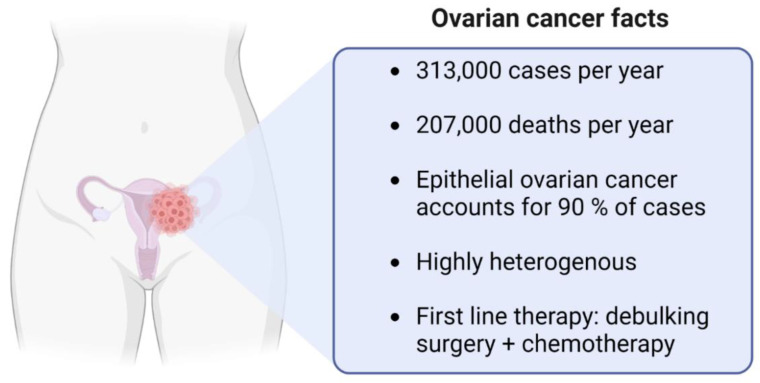
Ovarian cancer facts.

Ovarian cancer (OC) is the seventh deadliest and the eighth most common cancer in women, affecting 313,000 women and causing 207,000 deaths in 2020 (International Agency for Research on Cancer). OC is also called the “silent killer” because it is usually diagnosed at a late stage when the chances of a cure are already very low. Most OCs are diagnosed at stage III (51%) or IV (29%), where the 5-year survival is only 42% or 26%, respectively [1]. See Figure 1 for the most important facts of ovarian cancer. Ovarian tumors arise not only, as previously thought, in tissues of the ovary, but recent data shows that in some cases they can also start in the distal fallopian tube [2]. OC is a highly heterogeneous disease. Heterogeneity is high not only among the different types of ovarian tumors, but also within a single tumor [3]. Based on its origin, OC has seven histological types: epithelial tumors, mesenchymal tumors, mixed epithelial and mesenchymal tumors, sex cord stromal tumors, germ cell tumors miscellaneous tumors, and tumor-like lesions [4]. Around 90% of all ovarian tumors are of epithelial origin [5]. Because of their high heterogeneity, epithelial ovarian carcinomas (EOC) are divided into subgroups by the World Health Organization according to cell type: serous tumors, mucinous tumors, endometroid tumors, clear cell tumors, seromucinous tumors, Brenner tumors, and other carcinomas [4]. According to the International Federation of Gynecology and Obstetrics (FIGO), OC has four stages based on macroscopic and microscopic examination before and after surgery as well as cytology [6]. At stage I and II, the tumor is present mainly in ovaries and fallopian tubes, whereas at stage III it has spread already to local lymph nodes and peritoneum outside the pelvis. At the highest stage IV, distant metastases are present [7]. 

Treatment of OC can be local or systemic. Selection of the therapy depends on the type and stage of the disease. First-line therapy is usually debulking surgery and chemotherapy with platinum-based compounds and taxanes. There are also other, less common treatments such as radiation therapy, hormone therapy, but also targeted therapy, which is directed at genes and proteins specific for the cancers. The most common targeted therapy uses Poly(ADP-Ribose)-Polymerase (PARP) inhibitors. Unfortunately, current first-line chemotherapeutic treatments cause serious side effects such as dizziness, fatigue, nausea, vomiting, and diarrhea. That is why there is an urgent need to develop new adjuvant or curative therapeutic approaches that would decrease the required dose or duration of the classical chemotherapy, thus reducing side effect severity. 

## 2. Vitamin D and Vitamin D Analogs

Ultraviolet rays transform 7-dehydrocholesterol in the skin to cholecalciferol, or vitamin D3 [8]. Vitamin D is then transported to the liver where it is hydroxylated on position 25 to the main circulating metabolite 25-hydroxyvitamin D3 (25D3) by 25-hydroxylase encoded by the gene *CYP2R1*. 25D3, bound to the vitamin D binding protein, is transported to the target tissues, where it is hydroxylated on the position 1α to the main active hormone 1,25-dihydroxyvitamin D3, or calcitriol (1,25D3), by the rate-limiting enzyme 1α-hydroxylase, coded by the *CYP27B1* gene [8]. The kidneys are the main site for this hydroxylation step, but many other tissues express *CYP27B1* and thus are able to synthetize the active hormone 1,25D3. Both 25D3 and 1,25D3 are degraded by the enzyme 24-hydroxylase, encoded by *CYP24A1* [8]. The main role of 1,25D3, bound to its receptor, the transcription factor vitamin D receptor (VDR), is the regulation of serum calcium and phosphorus homeostasis, but it also regulates the expression of many genes that might play a role in the development of cancer [9]. The main indicator of the organism’s vitamin D status is serum 25D3, although the optimal status is still a subject of debate. Levels <12 ng/mL are considered as severe vitamin D deficiency, while sufficiency varies between 20-30 ng/mL, depending on the expert bodies or societies [10,11,12].

**Figure 2:**
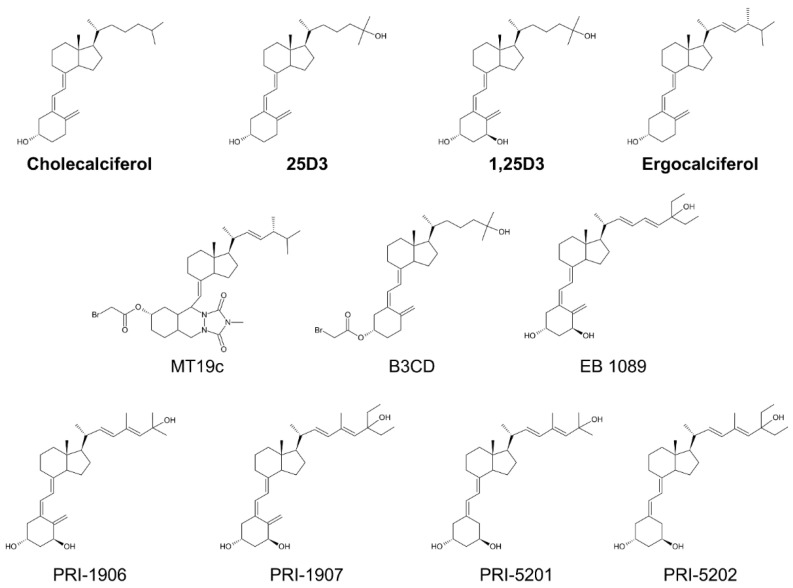
Chemical structures of the most important parent compounds (in bold) and the vitamin D analogs mentioned in the review.

The main function of 1,25D3 is to maintain the proper levels of calcium and phosphorus in serum. Thus, higher doses of 1,25D3, which would be effective against cancer, can cause hypercalcemia as a side effect. For this reason, various synthetic vitamin D analogs have been developed (representative structures of parent compounds and analogs are shown in Figure 2). MT19c is a vitamin D2 (the plant-derived ergocalciferol) derivate, created by Diels–Alder cycloaddition of *N*-methyl,1,2,4-triazolinedione and esterification with bromoacetic acid [13]. This last modification is shared with the analog B3Cd, which is a 3-bromoacetoxy derivative of 25D3 [14]. EB1089 was one of the first developed vitamin D analogs. It is based on the 1,25D3 structure by elongating the side chain by one carbon and the introduction of terminal ethyl groups and introduction of double bonds at positions 22 and 24 [15]. The PRI-1906 and PRI-1907 analogs were designed based on the 1,25-hydroxylated form (1,25D2) of the plant-derived vitamin D2 (ergocalciferol), with an extended side chain and introduced double bonds. PRI-1906 carries terminal methyl and PRI-1907 ethyl groups [16]. The analogs PRI-5201 and PRI-5202 are based on PRI-1906 and PRI-1907 by removing the methylidene group of the A-ring [17]. 

## 3. Effect of Vitamin D on Ovarian Cancer Epidemiology

Several recent studies reviewed the anticancer effects of different vitamins on selected female malignancies [18,19,20]; in our review, we focus only on the effect of vitamin D in ovarian cancer. The role of vitamin D levels in epidemiology of OC is still unclear. One of the first studies on the correlation between vitamin D and cancer found that sunlight can be a protective factor against OC-associated mortality [21]. A report from Australia concluded that exposure to ambient ultraviolet radiation may reduce the risk of EOC [22]. In another cohort study from Australia, researchers have shown that higher 25D3 serum levels at the stage of diagnosis correlated significantly with longer survival of women with diagnosed invasive OC [23]. In a European population, a Mendelian randomized study found that genetically lower 25D3 levels correlated inversely with higher susceptibility to OC [24]. Additionally, predicted higher concentrations of 25D3 (based on GWAS studies) were associated with reduced risk of EOC [25]. However, current data on this topic are inconsistent. In another Mendelian randomized study, researchers concluded that vitamin D levels had no effect in seven cancers, including OC [26]. A further study found that genetically low plasma 25D3 concentrations were not associated with increased cancer risk and mortality rates [27]. Similarly, a study conducted in African women from Nigeria found no significant correlation between serum 25D3 levels and the risk of EOC [28]. A meta-analysis of 21 articles with almost one million participants concluded that vitamin D intake could not decrease the risk of OC [29].

## 4. Mechanism of Anticancer Activity in Ovarian Cancer Models In Vitro

The heterogeneity of OC is mirrored by the diversity of the existing cell lines. Even those cell lines that were obtained from the same type of tumors show high diversity in their sensitivity to vitamin D and its analogs [30]. This might be due to differences in their mutational landscape, but also in the expression of the components of the vitamin D system. While VDR is present in most of the known cell lines, the expression is highly variable, as is also the expression of *CYP27B1* and *CYP24A1*. The results comparing the expression level of VDR in normal and malignant ovarian tissues are inconsistent. One study reported higher levels in ovarian tumors compared with healthy tissue [31], while others found that the VDR level was lower in tumors than in normal ovaries [32]. Treatment of OC cell lines with 1,25D3 or its analogs has no effect on VDR mRNA levels, while it might increase protein levels in a cell line-dependent manner [30,33]. Interestingly, the effect of 1,25D3 or its analogs on the expression of *CYP24A1* in different OC cell lines does not predict their anticancer effect [30].

### 4.1. Effect of 1,25D3 and Its Analogs on the Hallmarks of Cancer

**Figure 3:**
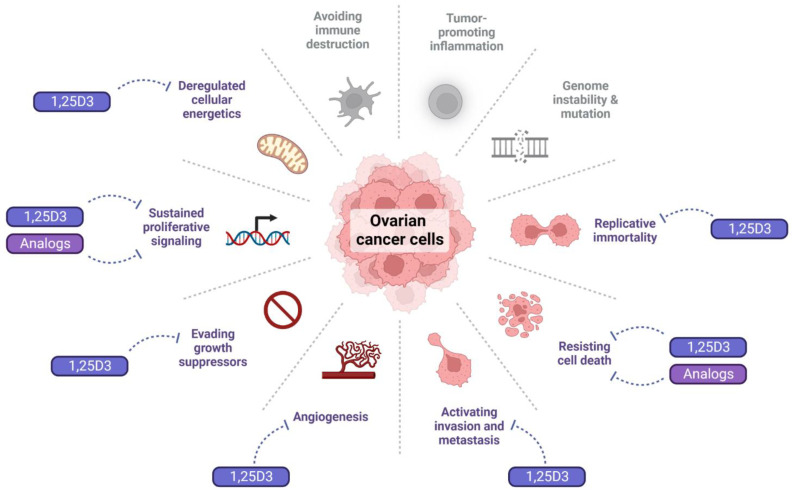
Impact of 1,25D3 and its analogs on the hallmarks of cancer in OC cells. In color, the hallmarks affected by 1,25D3 (see main text below), in gray, those where no relevant studies were found. The dotted blunt arrows indicate inhibition.

Besides its major role to maintain calcium-phosphate homeostasis, 1,25D3 regulates most hallmarks of cancer. It inhibits proliferation, angiogenesis, and metastasis, induces differentiation and apoptosis, and regulates the immune system [34,35]. The anticancer effects of 1,25D3 were best documented for colorectal, breast, and prostate cancer [36,37,38,39]. Much less is known about the effect of 1,25D3 and its analogs in OC cells (Figure 3).

### 4.2. Effect of 1,25D3 and Its Analogs on Proliferative Signals

1,25D3 inhibited proliferation by reducing the cell number of a patient-derived high grade serous ovarian cancer (HGSOC) cell line, while no effect was seen in a further HGSOC line [30]. In OVCAR3 cells, 1,25D3 induced cell cycle arrest either at the G1/S or G2/M checkpoint [40,41]. The expression of several genes involved in the cell cycle was also inhibited in some (e.g., OVCAR3, CAOV3, OV2008) but not in other cell lines (e.g., OVCAR5, SKOV3). Interestingly, another study found that SKOV3 cells were sensitive to 1,25D3, which reduced both their proliferation and viability [42]. In OVCAR3 cells, 1,25D3 prevented cell cycle progression through the inhibition of the CKD2-Rb-E2F axis. In these cells, 1,25D3 increased p27 and decreased cyclin E and A expression [40]. Proliferation of OVCAR3 and SKOV3 cells was inhibited also by the 25D3 analog B3CD [14].

Epidermal growth factor receptor (EGFR) is often upregulated in OC, conveying a proliferative advantage to these tumors [43,44]. In vitamin D-sensitive OC cells, 1,25D3 downregulated EGFR expression, reducing their proliferative potential [41]. The OVCAR3 cells were also sensitive to the 1,25D3 analog, EB1089, which was more active in reducing EGFR expression than 1,25D3 [41]. The relevance of the impact of vitamin D on EGFR is underlined also by the fact that EGFR seems to be one of the key genes associated with resistance to platinum therapy of OC [44].

We also observed that different HGSOC cell lines responded differently to various analogs of 1,25D2. While all tested analogs reduced cell number and viability of 13,781 cells, in the 14,433 and 8714 cells, none of the analogs affected cell viability significantly ([30] and unpublished data).

### 4.3. Effect of 1,25D3 and Its Analogs on Cell Death

Very often, p53 is mutated in ovarian tumors, reducing the apoptotic ability of these cells. Therefore, finding compounds that would induce apoptosis even in the presence of a mutated p53 is of utmost importance. Interestingly, 1,25D3 and the EB1089 analog were able to stimulate apoptosis through p53-independent ways, by upregulating Growth Arrest and DNA Damage-inducible 45 (GADD45), or the cyclin-dependent kinases p21 and p27 [45]. The pro-apoptotic role of GADD45 proteins is well documented and they regulate many cellular functions, e.g., DNA repair, cell cycle, and senescence [46]. In cell lines from clear cell ovarian carcinoma (ES-2, TOV-21G), papillary serous adenocarcinoma (OV-90) and endometrioid carcinoma (TOV-112D), 1,25D3 activated the intrinsic apoptotic pathway by reducing the membrane potential of the mitochondria, increasing cytochrome C release, and activating caspase 9 [47]. In the cell lines that expressed the progesterone receptor, the effect of 1,25D3 was significantly higher when given together with progesterone [48]. 

1,25D3 increased sensitivity of the ovarian epithelial adenocarcinoma cell line SKOV3 to radiation-induced apoptosis by supporting the formation of reactive oxygen species (ROS) [49]. Another study has shown that, in SKOV3 cells, 1,25D3 induced apoptosis and potentiated the cytotoxic effect of cisplatin, increasing the activity of caspase 3/7. It also increased expression of the pro-apoptotic protein Bax and the cleaved PARP in a dose-dependent manner [42]. B3CD induced apoptosis by activating the p38 MAPK pathway [14].

### 4.4. Effect of 1,25D3 and Its Analogs on Metastatic Potential

Epithelial to mesenchymal transition (EMT) is considered as the driver of invasion and metastasis. 1,25D3 inhibited EMT in SCOV3 cancer cells [50]. One of the first organs OC disseminates to is the peritoneum [51]. Vitamin D prevented the TGF-β-induced mesenchymal transition and thus the transformation of the peritoneal mesothelial cells into cancer-associated mesothelial cells (CAM) by maintaining high e-cadherin levels and blocking the upregulation of the EMT-associated markers α-smooth muscle actin, slug and the matrix metalloproteinases (MMP) 9 and 2, and that of thrombospondin-1, a gene involved in both the TGF-β and focal adhesion pathways [52]. Treatment of the germline-derived immortalized ovarian cancer cell line A2780 with 1,25D3 inhibited migration of the cells and their adhesion to fibronectin. Pre-treatment of the cells with 1,25D3 also reduced their metastatic potential when injected in immunodeficient mice [53]. 

Several long noncoding RNAs, e.g., lnc-BCAS1-4_1, play an important role in the regulation of EMT by 1,25D3 [54]. OC patients with high levels of the lncRNA *TOPORS Antisense RNA 1* (*TOPORS-AS1*) in their tumors had favorable overall survival compared with those expressing low levels. As *TOPORS-AS1* is a target for VDR, it has been suggested that the inhibitory effect of VDR in ovarian cancer cells could be mediated through *TOPORS-AS1* [55].

In a recent study, 1,25D3 inhibited the self-renewal capacity of ovarian cancer stem cells (CSC) by reduction of their sphere formation rate and inhibition of the expression of stem cell markers, such as CD44, SOX2, or OCT4 [56]. 

### 4.5. Effect of 1,25D3 and Its Analogs on Replicative Immortality and Angiogenesis

In the OVCAR3 cells, 1,25D3 inhibited a further hallmark of cancer, the replicative immortality, by downregulating activity and expression of the telomerase [57]. One mechanism by which 1,25D3 regulated telomerase expression in these cells was the upregulation of miR-498, which then degrades the telomerase mRNA, leading to their apoptotic cell death [58].

In SKOV3, 1,25D3 inhibited VEGF expression and activity and enhanced the anti-angiogenic effect of cisplatin [42].

There is not enough information on the role that vitamin D could play in regulating other hallmarks. Although it is known that vitamin D affects the immune system and plays an important role in inflammation, little is known about its effect on these hallmarks in OC. One study has shown that 1,25D3 was able to reduce the tumor promoting effect of M2 macrophages in ovarian cancer cells [59]. Another interesting finding was that the vitamin D target human cathelicidin, well known as an effector molecule of the innate immune system, promotes OC progression. It seems that OC cells induce the expression of cathelicidin in macrophages in a VDR-dependent manner [60]. This would suggest a detrimental effect of vitamin D on OC development. 

## 5. Mechanism of Anticancer Activity in Ovarian Cancer Models In Vivo

**Figure 4:**
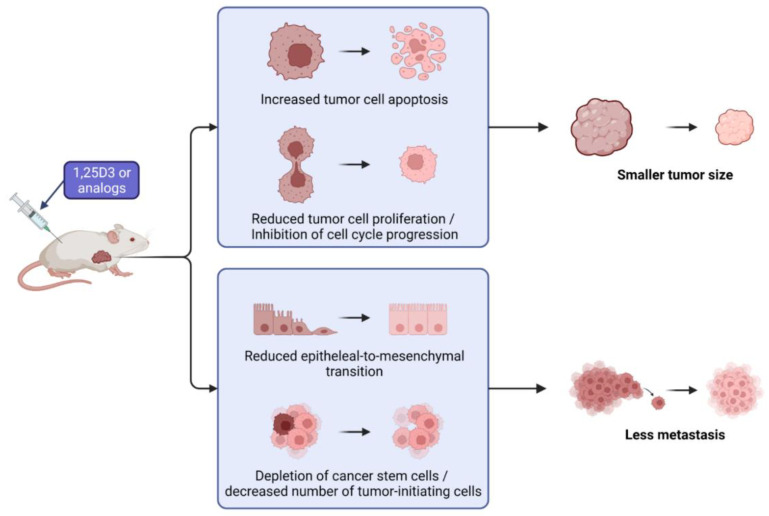
Effects of 1,25D3 and its analogs in in vivo models of ovarian cancer.

The anticancer activity of 1,25D3 and its analogs in OC models has already been studied broadly in vitro but there are only a few studies about their effects in vivo. Figure 4 summarizes the observed effects found in in vivo studies.

In a mouse model of peritoneal metastasis, vitamin D3 protected the microvilli on the peritoneum, thus preventing the interaction of CAMs with cancer cells by inhibiting Smad-dependent TGF-β signaling, thus inhibiting peritoneal dissemination of the ES-2 OC cells [52].

Vitamin D affects at different stages of the carcinogenesis process. In a mouse model of OC, induced by 7,12-dimethylbenz[a]anthracene (DMBA), 1,25D3 administration reduced tumor size significantly at the stage of initiation, promotion and entire period of the experiment. The general condition of the mice in the treated groups was significantly better than in the untreated controls [61]. Mice treated with vitamin 1,25D3 also had lower levels of CA125, which is considered a potential ovarian tumor marker [61,62].

1,25D3 as an anticancer agent is known to also regulate CSCs. Srivastava et al. studied the effect of 1,25D3 on OC stem cells in vivo in a mouse xenograft tumor model using the 2008 cell line. 1,25D3 delayed tumor growth and depleted the ovarian CSCs. The effect on the CSCs was studied in isolated xenograft tumor cells by measuring CD44 and CD117 positive cells, which are markers for stem cells. 1,25D3 significantly reduced the CSC population in ovarian xenografts in vivo [63].

Only a few analogs of vitamin D3 or 25D3 were tested in vivo in ovarian cancer models. MT19c reduced tumor growth in the SKOV3 xenograft model in nude mice and in a syngeneic rat ovarian cancer models and decreased the expression of genes involved in energy metabolism. The treatment reduced the expression of EGFR, and inhibited PI-3 kinase [13]. The 25D3 derivative B3CD was tested on the SKOV-3 xenograft model. The compound delayed tumor growth in the majority of the mice, and in some cases even led to full regression. However, in some of the mice, B3CD accelerated tumor growth [14]. The study with the 1,25D3 analog EB 1089 found that EB 1089 suppressed the growth of OVCAR3 tumor xenografts in mice. Histological analysis of tumor sections showed that EB 1089 induced apoptosis and decreased proliferation in the tumor [45]. 

More in vivo studies are needed to understand if vitamin D analogs should be carried further into clinical trials. 

## 6. Future Perspectives

The impact of vitamin D and its analogs on OC is still unclear, although the in vitro studies are promising. Further to the research we summarized, a few studies reported that 1,25D3 is able to potentiate the effect of some of the chemotherapeutics used in the treatment of OC. 1,25D3 increased the cytotoxic effect of carboplatin and paclitaxel in serous-, mucinous-, and endometrioid-type OC cell lines [33]. Although it has been shown that 1,25D3 is a PARP inhibitor [64], the studies to test if it would potentiate the effect of the PARP inhibitors used in OC treatment are still missing. PARP inhibitors are used for the therapy of recurrent OC, however, the majority of the patients develop resistance, mediated by cancer stem cells [65]. As 1,25D3 was shown to reduce the number of CSCs in OC [63], this suggests a high clinical potential in preventing resistance to this class of drugs. Therefore, it would be of utmost importance to understand what makes OC cells responsive or resistant to the anticancer effects of vitamin D and its analogs. More needs to be done especially in vivo to gain a clear picture about the impact and potential application of vitamin D and its less calcemic analogs in OC.

## Data Availability

Not applicable.

## References

[1] Torre L.A., Trabert B., DeSantis C.E., Miller K.D., Samimi G., Runowicz C.D., Gaudet M.M., Jemal A., Siegel R.L. (2018). Ovarian cancer statistics, 2018. CA Cancer J. Clin..

[2] Ducie J., Dao F., Considine M., Olvera N., Shaw P.A., Kurman R.J., Shih I.M., Soslow R.A., Cope L., Levine D.A. (2017). Molecular analysis of high-grade serous ovarian carcinoma with and without associated serous tubal intra-epithelial carcinoma. Nat. Commun..

[3] Kossai M., Leary A., Scoazec J.Y., Genestie C. (2018). Ovarian Cancer: A Heterogeneous Disease. Pathobiology.

[4] Adhikari L.H.L. Ovarian Neoplasms WHO Classification Review. https://www.pathologyoutlines.com/topic/ovarytumorwhoclassif.html.

[5] Reid B.M., Permuth J.B., Sellers T.A. (2017). Epidemiology of ovarian cancer: A review. Cancer Biol. Med..

[6] Matulonis U.A., Sood A.K., Fallowfield L., Howitt B.E., Sehouli J., Karlan B.Y. (2016). Ovarian cancer. Nat. Rev. Dis. Primers.

[7] Berek J.S., Crum C., Friedlander M. (2012). Cancer of the ovary, fallopian tube, and peritoneum. Int. J. Gynaecol. Obstet..

[8] Bouillon R., Carmeliet G., Verlinden L., van Etten E., Verstuyf A., Luderer H.F., Lieben L., Mathieu C., Demay M. (2008). Vitamin D and human health: Lessons from vitamin D receptor null mice. Endocr. Rev..

[9] Carlberg C., Munoz A. (2022). An update on vitamin D signaling and cancer. Semin. Cancer Biol..

[10] Amrein K., Scherkl M., Hoffmann M., Neuwersch-Sommeregger S., Kostenberger M., Tmava Berisha A., Martucci G., Pilz S., Malle O. (2020). Vitamin D deficiency 2.0: An update on the current status worldwide. Eur. J. Clin. Nutr..

[11] Holick M.F., Binkley N.C., Bischoff-Ferrari H.A., Gordon C.M., Hanley D.A., Heaney R.P., Murad M.H., Weaver C.M., Endocrine S. (2011). Evaluation, treatment, and prevention of vitamin D deficiency: An Endocrine Society clinical practice guideline. J. Clin. Endocrinol. Metab..

[12] Bresson J.L., Burlingame B., Dean T., Fairweather-Tait S., Heinonen M., Hirsch-Ernst K.I., Mangelsdorf I., McArdle H., Naska A., Neuhauser-Berthold M. (2016). Dietary reference values for vitamin D. Efsa J..

[13] Moore R.G., Lange T.S., Robinson K., Kim K.K., Uzun A., Horan T.C., Kawar N., Yano N., Chu S.R., Mao Q. (2012). Efficacy of a non-hypercalcemic vitamin-D2 derived anti-cancer agent (MT19c) and inhibition of fatty acid synthesis in an ovarian cancer xenograft model. PLoS ONE.

[14] Lange T.S., Stuckey A.R., Robison K., Kim K.K., Singh R.K., Raker C.A., Brard L. (2010). Effect of a vitamin D(3) derivative (B3CD) with postulated anti-cancer activity in an ovarian cancer animal model. Invest. New Drugs.

[15] Davicco M.J., Coxam V., Gaumet N., Lebecque P., Barlet J.P. (1995). EB 1089, a calcitriol analogue, decreases fetal calcium content when injected into pregnant rats. Exp. Physiol..

[16] Baurska H., Klopot A., Kielbinski M., Chrobak A., Wijas E., Kutner A., Marcinkowska E. (2011). Structure-function analysis of vitamin D(2) analogs as potential inducers of leukemia differentiation and inhibitors of prostate cancer proliferation. J. Steroid Biochem. Mol. Biol..

[17] Pietraszek A., Malinska M., Chodynski M., Krupa M., Krajewski K., Cmoch P., Wozniak K., Kutner A. (2013). Synthesis and crystallographic study of 1,25-dihydroxyergocalciferol analogs. Steroids.

[18] Markowska A., Antoszczak M., Markowska J., Huczynski A. (2022). Role of Vitamin K in Selected Malignant Neoplasms in Women. Nutrients.

[19] Markowska A., Antoszczak M., Markowska J., Huczynski A. (2022). Role of Vitamin E in Selected Malignant Neoplasms in Women. Nutr. Cancer.

[20] Markowska A., Antoszczak M., Markowska J., Huczynski A. (2022). Role of Vitamin C in Selected Malignant Neoplasms in Women. Nutrients.

[21] Lefkowitz E.S., Garland C.F. (1994). Sunlight, vitamin D, and ovarian cancer mortality rates in US women. Int. J. Epidemiol..

[22] Tran B., Jordan S.J., Lucas R., Webb P.M., Neale R., Australian Ovarian Cancer Study Group (2012). Association between ambient ultraviolet radiation and risk of epithelial ovarian cancer. Cancer Prev. Res. (Phila).

[23] Webb P.M., de Fazio A., Protani M.M., Ibiebele T.I., Nagle C.M., Brand A.H., Blomfield P.I., Grant P., Perrin L.C., Neale R.E. (2015). Circulating 25-hydroxyvitamin D and survival in women with ovarian cancer. Am. J. Clin. Nutr..

[24] Ong J.S., Cuellar-Partida G., Lu Y., Fasching P.A., Hein A., Burghaus S., Beckmann M.W., Lambrechts D., Van Nieuwenhuysen E., Vergote I. (2016). Association of vitamin D levels and risk of ovarian cancer: A Mendelian randomization study. Int. J. Epidemiol..

[25] Ong J.S., Dixon-Suen S.C., Han X., An J., Liyanage U., Me Research T., Dusingize J.C., Schumacher J., Gockel I., Böhmer A. (2021). A comprehensive re-assessment of the association between vitamin D and cancer susceptibility using Mendelian randomization. Nat. Commun..

[26] Dimitrakopoulou V.I., Tsilidis K.K., Haycock P.C., Dimou N.L., Al-Dabhani K., Martin R.M., Lewis S.J., Gunter M.J., Mondul A., Shui I.M. (2017). Circulating vitamin D concentration and risk of seven cancers: Mendelian randomisation study. BMJ.

[27] Ong J.S., Gharahkhani P., An J., Law M.H., Whiteman D.C., Neale R.E., MacGregor S. (2018). Vitamin D and overall cancer risk and cancer mortality: A Mendelian randomization study. Hum. Mol. Genet..

[28] Sajo E.A., Okunade K.S., Olorunfemi G., Rabiu K.A., Anorlu R.I. (2020). Serum vitamin D deficiency and risk of epithelial ovarian cancer in Lagos, Nigeria. Ecancermedicalscience.

[29] Xu J., Chen K., Zhao F., Huang D., Zhang H., Fu Z., Xu J., Wu Y., Lin H., Zhou Y. (2021). Association between vitamin D/calcium intake and 25-hydroxyvitamin D and risk of ovarian cancer: A dose-response relationship meta-analysis. Eur. J. Clin. Nutr..

[30] Piatek K., Kutner A., Cacsire Castillo-Tong D., Manhardt T., Kupper N., Nowak U., Chodynski M., Marcinkowska E., Kallay E., Schepelmann M. (2021). Vitamin D Analogs Regulate the Vitamin D System and Cell Viability in Ovarian Cancer Cells. Int. J. Mol. Sci..

[31] Friedrich M., Rafi L., Mitschele T., Tilgen W., Schmidt W., Reichrath J. (2003). Analysis of the vitamin D system in cervical carcinomas, breast cancer and ovarian cancer. Recent. Results Cancer Res..

[32] Brozyna A.A., Kim T.K., Zablocka M., Jozwicki W., Yue J., Tuckey R.C., Jetten A.M., Slominski A.T. (2020). Association among Vitamin D, Retinoic Acid-Related Orphan Receptors, and Vitamin D Hydroxyderivatives in Ovarian Cancer. Nutrients.

[33] Kuittinen T., Rovio P., Luukkaala T., Laurila M., Grenman S., Kallioniemi A., Maenpaa J. (2020). Paclitaxel, Carboplatin and 1,25-D3 Inhibit Proliferation of Ovarian Cancer Cells In Vitro. Anticancer Res..

[34] Wacker M., Holick M.F. (2013). Vitamin D—Effects on skeletal and extraskeletal health and the need for supplementation. Nutrients.

[35] Feldman D., Krishnan A.V., Swami S., Giovannucci E., Feldman B.J. (2014). The role of vitamin D in reducing cancer risk and progression. Nat. Rev. Cancer.

[36] Ferrer-Mayorga G., Larriba M.J., Crespo P., Munoz A. (2019). Mechanisms of action of vitamin D in colon cancer. J. Steroid Biochem Mol. Biol..

[37] Mahendra A., Choudhury B.K., Sharma T., Bansal N., Bansal R., Gupta S. (2018). Vitamin D and gastrointestinal cancer. J. Lab. Physicians.

[38] Vanhevel J., Verlinden L., Doms S., Wildiers H., Verstuyf A. (2022). The role of vitamin D in breast cancer risk and progression. Endocr. Relat. Cancer.

[39] Swami S., Krishnan A.V., Feldman D. (2011). Vitamin D metabolism and action in the prostate: Implications for health and disease. Mol. Cell Endocrinol..

[40] Li P., Li C., Zhao X., Zhang X., Nicosia S.V., Bai W. (2004). p27(Kip1) stabilization and G(1) arrest by 1,25-dihydroxyvitamin D(3) in ovarian cancer cells mediated through down-regulation of cyclin E/cyclin-dependent kinase 2 and Skp1-Cullin-F-box protein/Skp2 ubiquitin ligase. J. Biol. Chem..

[41] Shen Z., Zhang X., Tang J., Kasiappan R., Jinwal U., Li P., Hann S., Nicosia S.V., Wu J., Zhang X. (2011). The coupling of epidermal growth factor receptor down regulation by 1alpha,25-dihydroxyvitamin D3 to the hormone-induced cell cycle arrest at the G1-S checkpoint in ovarian cancer cells. Mol. Cell Endocrinol..

[42] Kim J.H., Park W.H., Suh D.H., Kim K., No J.H., Kim Y.B. (2021). Calcitriol Combined With Platinum-based Chemotherapy Suppresses Growth and Expression of Vascular Endothelial Growth Factor of SKOV-3 Ovarian Cancer Cells. Anticancer Res..

[43] Olbromski P.J., Pawlik P., Bogacz A., Sajdak S. (2022). Identification of New Molecular Biomarkers in Ovarian Cancer Using the Gene Expression Profile. J. Clin. Med..

[44] Han L., Guo X., Du R., Guo K., Qi P., Bian H. (2022). Identification of key genes and pathways related to cancer-associated fibroblasts in chemoresistance of ovarian cancer cells based on GEO and TCGA databases. J. Ovarian Res..

[45] Zhang X., Jiang F., Li P., Li C., Ma Q., Nicosia S.V., Bai W. (2005). Growth suppression of ovarian cancer xenografts in nude mice by vitamin D analogue EB1089. Clin. Cancer Res..

[46] Tamura R.E., de Vasconcellos J.F., Sarkar D., Libermann T.A., Fisher P.B., Zerbini L.F. (2012). GADD45 proteins: Central players in tumorigenesis. Curr. Mol. Med..

[47] McGlorthan L., Paucarmayta A., Casablanca Y., Maxwell G.L., Syed V. (2021). Progesterone induces apoptosis by activation of caspase-8 and calcitriol via activation of caspase-9 pathways in ovarian and endometrial cancer cells in vitro. Apoptosis.

[48] Rodriguez G.C., Turbov J., Rosales R., Yoo J., Hunn J., Zappia K.J., Lund K., Barry C.P., Rodriguez I.V., Pike J.W. (2016). Progestins inhibit calcitriol-induced CYP24A1 and synergistically inhibit ovarian cancer cell viability: An opportunity for chemoprevention. Gynecol. Oncol..

[49] Ji M.T., Nie J., Nie X.F., Hu W.T., Pei H.L., Wan J.M., Wang A.Q., Zhou G.M., Zhang Z.L., Chang L. (2020). 1alpha,25(OH)2D3 Radiosensitizes Cancer Cells by Activating the NADPH/ROS Pathway. Front. Pharmacol..

[50] Hou Y.F., Gao S.H., Wang P., Zhang H.M., Liu L.Z., Ye M.X., Zhou G.M., Zhang Z.L., Li B.Y. (2016). 1alpha,25(OH)(2)D(3) Suppresses the Migration of Ovarian Cancer SKOV-3 Cells through the Inhibition of Epithelial-Mesenchymal Transition. Int. J. Mol. Sci..

[51] Siegel R.L., Miller K.D., Fuchs H.E., Jemal A. (2021). Cancer Statistics, 2021. CA Cancer J. Clin..

[52] Kitami K., Yoshihara M., Tamauchi S., Sugiyama M., Koya Y., Yamakita Y., Fujimoto H., Iyoshi S., Uno K., Mogi K. (2022). Peritoneal Restoration by Repurposing Vitamin D Inhibits Ovarian Cancer Dissemination via Blockade of the TGF-beta1/Thrombospondin-1 Axis. Matrix Biol..

[53] Abdelbaset-Ismail A., Pedziwiatr D., Suszynska E., Sluczanowska-Glabowska S., Schneider G., Kakar S.S., Ratajczak M.Z. (2016). Vitamin D3 stimulates embryonic stem cells but inhibits migration and growth of ovarian cancer and teratocarcinoma cell lines. J. Ovarian Res..

[54] Xue Y., Wang P., Jiang F., Yu J., Ding H., Zhang Z., Pei H., Li B. (2021). A Newly Identified lncBCAS1-4_1 Associated With Vitamin D Signaling and EMT in Ovarian Cancer Cells. Front. Oncol..

[55] Fu Y., Katsaros D., Biglia N., Wang Z., Pagano I., Tius M., Tiirikainen M., Rosser C., Yang H., Yu H. (2021). Vitamin D receptor upregulates lncRNA TOPORS-AS1 which inhibits the Wnt/beta-catenin pathway and associates with favorable prognosis of ovarian cancer. Sci. Rep..

[56] Ji M., Liu L., Hou Y., Li B. (2019). 1alpha,25Dihydroxyvitamin D3 restrains stem celllike properties of ovarian cancer cells by enhancing vitamin D receptor and suppressing CD44. Oncol. Rep..

[57] Jiang F., Bao J., Li P., Nicosia S.V., Bai W. (2004). Induction of ovarian cancer cell apoptosis by 1,25-dihydroxyvitamin D3 through the down-regulation of telomerase. J. Biol. Chem..

[58] Kasiappan R., Shen Z., Tse A.K., Jinwal U., Tang J., Lungchukiet P., Sun Y., Kruk P., Nicosia S.V., Zhang X. (2012). 1,25-Dihydroxyvitamin D3 suppresses telomerase expression and human cancer growth through microRNA-498. J. Biol. Chem..

[59] Guo Y., Jiang F., Yang W., Shi W., Wan J., Li J., Pan J., Wang P., Qiu J., Zhang Z. (2022). Effect of 1alpha,25(OH)2D3-Treated M1 and M2 Macrophages on Cell Proliferation and Migration Ability in Ovarian Cancer. Nutr. Cancer.

[60] Li D., Wang X., Wu J.L., Quan W.Q., Ma L., Yang F., Wu K.Y., Wan H.Y. (2013). Tumor-produced versican V1 enhances hCAP18/LL-37 expression in macrophages through activation of TLR2 and vitamin D3 signaling to promote ovarian cancer progression in vitro. PLoS ONE.

[61] Liu L., Hu Z., Zhang H., Hou Y., Zhang Z., Zhou G., Li B. (2016). Vitamin D postpones the progression of epithelial ovarian cancer induced by 7, 12-dimethylbenz [a] anthracene both in vitro and in vivo. Onco Targets Ther..

[62] Gandhi T., Bhatt H. (2022). Cancer Antigen 125.

[63] Srivastava A.K., Rizvi A., Cui T., Han C., Banerjee A., Naseem I., Zheng Y., Wani A.A., Wang Q.E. (2018). Depleting ovarian cancer stem cells with calcitriol. Oncotarget.

[64] Rizvi A., Naseem I. (2020). Causing DNA damage and stopping DNA repair—Vitamin D supplementation with Poly(ADP-ribose) polymerase 1 (PARP1) inhibitors may cause selective cell death of cancer cells: A novel therapeutic paradigm utilizing elevated copper levels within the tumour. Med. Hypotheses.

[65] Bellio C., DiGloria C., Foster R., James K., Konstantinopoulos P.A., Growdon W.B., Rueda B.R. (2019). PARP Inhibition Induces Enrichment of DNA Repair-Proficient CD133 and CD117 Positive Ovarian Cancer Stem Cells. Mol. Cancer Res..

